# Meta‐analysis of NIH Alzheimer’s Disease‐Related Dementias (ADRD) programs responsive to the National Plan to Address Alzheimer’s Disease (AD)

**DOI:** 10.1002/alz.089760

**Published:** 2025-01-03

**Authors:** Amber J McCartney, Herson Astacio, Arvind Shukla, Xiling Yin, Sara Dodson, Erin Bryant, Roderick A Corriveau

**Affiliations:** ^1^ National Institutes of Health (NIH)/National Institute of Neurological Disorders and Stroke (NINDS), Bethesda, MD USA

## Abstract

**Background:**

AD/ADRD diseases currently impact more than 6 million people in the US. Rare forms of AD/ADRD are caused directly and unambiguously by genetic mutations. However, most AD/ADRD burden is complex in etiology and thought to result from an interplay among multiple incompletely understood genetic, biochemical, lifestyle, environmental and psychosocial risk factors. Moreover, risk factors interact with other health factors to impact AD/ADRD outcomes. For example, while the most common dementia diagnosis is AD, research over the past decade revealed most people with a diagnosis of AD have multiple brain pathologies, other comorbidities, and many people diagnosed with clinical AD do not have AD pathology at all. This new knowledge highlights the importance of better understanding dementia syndromes, and the relationships among them, to increase chances of developing effective interventions. The NINDS partners with the NIA, the NIH lead for AD/ADRD, in the NIH response to the National Plan. NINDS leads ADRD including frontotemporal dementia (FTD), Lewy body dementias (LBD), vascular contributions to cognitive impairment and dementia (VCID) and multiple etiology dementias (MED). This project aims to evaluate NIH and the ADRD field’s responsiveness to ADRD research milestones from triennial NINDS ADRD Summits by mapping NIH‐funded research activities to ADRD milestones.

**Method:**

The approach used text‐based analysis/mining of grant titles, abstracts, and specific aims to map awards and activities to ADRD milestones. Mappings were vetted by expert concurrence. The ongoing study includes new or competitively renewed NIH ADRD awards starting from fiscal year 2017. Findings help NIH to better understand the state of the field, identify research gaps and opportunities for future efforts, and enable public communication of research activities with an accurate, accessible, compact, and updatable data visualizations.

**Result:**

Results indicate the approach is feasible, and importantly the field and NIH have largely been responsive to the ADRD milestones in the National Plan to Address AD.

**Conclusion:**

The analysis points to several cross‐cutting ADRD research areas that may benefit from increased attention going forward, including health equity, the multiple etiology hypothesis of common dementias, emerging risk factors for common dementias (e.g. COVID‐19, TBI, and TDP‐43 proteinopathy), and clinical trials.

